# Impact of Varying Light and Dew on Ground Cover Estimates from Active NDVI, RGB, and LiDAR

**DOI:** 10.34133/2021/9842178

**Published:** 2021-05-26

**Authors:** David M. Deery, David J. Smith, Robert Davy, Jose A. Jimenez-Berni, Greg J. Rebetzke, Richard A. James

**Affiliations:** ^1^CSIRO Agriculture and Food, Canberra, ACT, Australia; ^2^CSIRO Agriculture and Food, Yanco, NSW, Australia; ^3^CSIRO Information Management and Technology, Canberra, ACT, Australia; ^4^Instituto Agricultura Sostenible, Consejo Superior de Investigaciones Cientificas, Cordoba, Spain

## Abstract

Canopy ground cover (GC) is an important agronomic measure for evaluating crop establishment and early growth. This study evaluates the reliability of GC estimates, in the presence of varying light and dew on leaves, from three different ground-based sensors: (1) normalized difference vegetation index (NDVI) from the commercially available GreenSeeker®; (2) RGB images from a digital camera, where GC was determined as the portion of pixels from each image meeting a greenness criterion (i.e., (Green − Red)/(Green + Red) > 0); and (3) LiDAR using two separate approaches: (a) GC from LiDAR red reflectance (whereby red reflectance less than five was classified as vegetation) and (b) GC from LiDAR height (whereby height greater than 10 cm was classified as vegetation). Hourly measurements were made early in the season at two different growth stages (tillering and stem elongation), among wheat genotypes highly diverse for canopy characteristics. The active NDVI showed the least variation through time and was particularly stable, regardless of the available light or the presence of dew. In addition, between-sample-time Pearson correlations for NDVI were consistently high and significant (*P* < 0.0001), ranging from 0.89 to 0.98. In comparison, GC from LiDAR and RGB showed greater variation across sampling times, and LiDAR red reflectance was strongly influenced by the presence of dew. Excluding times when the light was exceedingly low, correlations between GC from RGB and NDVI were consistently high (ranging from 0.79 to 0.92). The high reliability of the active NDVI sensor potentially affords a high degree of flexibility for users by enabling sampling across a broad range of acceptable light conditions.

## 1. Introduction

Canopy ground cover (GC) represents the proportion of the soil surface covered by plant foliage and is an important measure for characterising crop establishment and early crop growth. The latter is often termed early vigour. The GC is therefore related to the rate of development of above-ground biomass (AGB) and leaf area index (LAI), defined as the area of foliage per unit of land area. Conventional methods for the precise phenotyping of early vigour have included destructive sampling to determine AGB and/or LAI. Additionally, the leaf area-to-leaf width ratio, known as the specific leaf area (SLA), of seedling leaves has been used as a predictor of early vigour [1]. However, such destructive methods are generally considered too labour-intensive and time-consuming for screening many genotypes in large experiments or within plant breeding programs.

Genotypes with greater early GC generally intercept more radiation (necessary for growth) and shade a greater proportion of the soil, thereby reducing soil evaporation and potentially increasing water-use efficiency [2–5]. However, the trait is of potentially greater benefit in wetter growing environments [6], with the risk in arid environments that too much early growth will exhaust soil water, resulting in greater terminal drought stress [7]. Nevertheless, high GC can result in greater weed competitiveness [8] and thereby potentially assist with the management of herbicide-resistant weed populations [9].

The GC is generally measured from crop emergence until the canopy reaches full cover. Methods for measuring GC typically involve either digital photographs (e.g., RGB images and multispectral cameras) or dedicated sensors for quantifying spectral indices. In the former, RGB images are processed to classify the proportion of vegetation and nonvegetation pixels [5, 10–14]. There are various classification techniques for distinguishing between vegetative and nonvegetative pixels. These range from the rather straightforward, but generally effective, approach of identifying the portion of green pixels in a given image [11] to more complex image analysis methods, including machine learning [15, 16]. A commonly reported spectral index for measuring GC is the normalized difference vegetation index (NDVI) which is the near-infrared radiation minus red radiation divided by the sum of near-infrared radiation and red radiation [17]. It is possible to derive the NDVI using passive or active sensing [18]. In this context, sensors are distinguished between those that provide their own light source, known as active sensors, and those that rely on ambient light, known as passive sensors. Therefore, the active sensors, which concomitantly produce light in particular spectral regions, are likely to operate more independently of the ambient light conditions than passive sensors. Moreover, active sensors are generally limited to measuring a few discrete wavelengths, dictated by their light source. In contrast, passive sensors typically measure across a broader range of wavelengths, enabling derivation of various vegetation indices, and are therefore generally more flexible and able to service a broad range of applications. Passive sensors do, however, require suitable light conditions.

Light detection and ranging (LiDAR), a laser-based remote sensing technology that primarily measures distance, has been proposed for field phenotyping [19]. LiDAR mounted on a portable terrestrial phenotyping platform (“Phenomobile Lite™”) was recently proposed for nondestructive assessment of canopy height, GC, and above-ground biomass [14, 20–22]. Jimenez-Berni and colleagues [14] developed two LiDAR algorithms for the estimation of GC that were shown to be highly correlated with GC derived from RGB images and NDVI measured using the commercially available GreenSeeker® sensor: GC from LiDAR red reflectance (GC_LiDAR_^RR^) and GC from LiDAR height (GC_LiDAR_^HT^). The GC_LiDAR_^RR^ assumes that green plant tissue will absorb more of the LiDAR's red laser than soil. The vegetation and soil are typically evident as two distinct peaks in the histogram analysis of the LiDAR red reflectance (refer to Figure 5 in [14]). The histogram peaks were used to estimate GC_LiDAR_^RR^, whereby LiDAR intensity less than five was classified as vegetation and LiDAR intensity of five and above was classified as soil (refer to Figures 6a,b in [14]). The GC_LiDAR_^HT^ is based on the premise that because the LiDAR creates a three-dimensional representation of the canopy, any organ or tissue above the ground can be considered vegetation, and therefore, the determination of GC can be derived from this classification (refer Figures 6c,d in [14]).

The present study is motivated by the need to reliably phenotype many genotypes for GC in large experiments or within plant breeding programs. Reliability in this context extends to performance under a range of weather conditions and times of day. As highlighted previously [23], this application requires the capacity to consistently discriminate varying levels of trait expression at a suitable level of repeatability across large numbers of genotypes. In this paper, concurrent GC measurements were derived from an active NDVI GreenSeeker® sensor, RGB (GC_RGB_), and LiDAR (GC_LiDAR_^RR^ and GC_LiDAR_^HT^). Measurements were made early in the season at hourly intervals over the course of two consequtive days, and conditions varied for intensity of both light and moisture (dry and with dew evident). The goal of this study was to evaluate the efficacy of these different methods for determining GC estimates early in the season, across a range of light conditions and for varying leaf moisture conditions. Specifically, this study is aimed at testing the hypothesis that the GC measurements derived from the active methods (NDVI and LiDAR) are more reliable than those from RGB when light and moisture conditions are not optimal (i.e., full sunlight and dry canopy). An additional consideration in this study is the contrasting measurement approach between the NDVI GreenSeeker® sensor and the GC estimates derived from the RGB and LiDAR. The NDVI obtained from the GreenSeeker® sensor is proportional to the difference in reflectance at two different spectral bands (near-infrared and red), whereby the reflectance is integrated over the area sampled (i.e., a band ratio). In contrast, the GC estimates determined from the RGB and LiDAR are both reliant on the thresholding of pixels over the area imaged (i.e., a pixel ratio).

## 2. Material and Methods

### 2.1. Field Experiment

A field experiment was established in 2017 at the Managed Environment Facility (MEF) [24], located at Yanco Agricultural Institute (34.62°S, 146.43°E, elevation 164 m) in south-eastern Australia. The soil at the Yanco MEF is classified as chromosol and has a clay-loam texture [25]. The experiment was sown on the 29^th^ of May in 2017 following a field pea break-crop and then managed with adequate nutrition and chemical controls as required for pest, weed, and leaf diseases. The experiment comprised 192 experimental plots, 6 m long containing seven rows of 25 cm spacing (orientated North-South), sowing density of 200 seeds per m^2^, and paths between plots of ca. 0.4 m.

The germplasm in the experiment comprised 99 wheat genotypes representing a series of near-isogenic lines varying for a range of canopy architecture traits including plant height, tiller number, leaf waxiness, plant development, and canopy erectness (as confirmed in prior studies [21, 22]). The genotypes were sown into a partial-replicate design experiment with the genotype replication averaging 1.9.

Meteorological data was obtained from the Bureau of Meteorology (http://www.bom.gov.au) weather station located adjacent the experiment site at the Yanco Agricultural Institute (station number 074037) (Table 1). As a relative indicator of the diurnal variation in daylight, solar radiation was measured at Griffith NSW (ca. 60 km north-west from the experiment site), being the closest measurement of solar radiation to the experimental site.

### 2.2. Data Acquisition

#### 2.2.1. Phenomobile Lite

**Figure 1:**
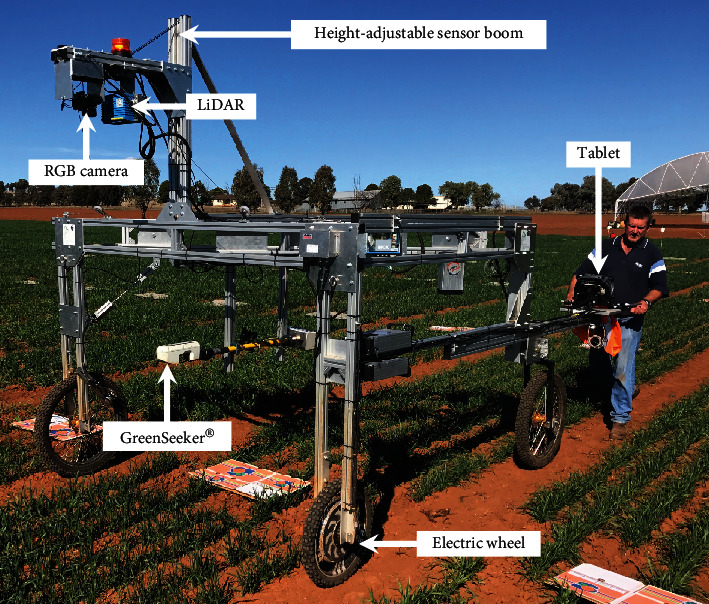
Phenomobile Lite™ comprising LiDAR, RGB camera, GreenSeeker®, and tablet. The boxes on the ground were used to demarcate the locations of the start and end of the individual plots when processing the data. The Phenomobile Lite traversed the experiment in a serpentine fashion, along the same direction as the sown rows (north-south orientation).

Data was acquired with the previously described Phenomobile Lite™ [14] (Figure 1). The Phenomobile Lite is a portable terrestrial phenotyping platform, which is manually steered and powered by an electric wheel. It comprises a lightweight extruded aluminum frame with three wheels containing the following instruments: (1) a high-frequency laser scanner or LiDAR (SICK LMS400, Waldkirch, Germany, for which the technical specifications are 70° field of view; monochromatic laser 650 nm, 4.0-7.5 mW; 0.7-3.0 m range; 1 mm distance resolution; scanning rate set to 270 Hz; and angular resolution of 0.1°), (2) an active NDVI GreenSeeker® sensor (Trimble, Sunnyvale, California, USA) that acquired data at 10 Hz, and (3) a digital camera (Canon 6D, Canon Inc., Tokyo, Japan) that was triggered by the control software to acquire an image every meter. Although the possible range of NDVI values extends from -1 to 1, the NDVI GreenSeeker® values range from 0 to 0.99 [26]. While it is possible to obtain a negative NDVI (i.e., red reflectance > near‐infrared reflectance), this is highly implausible for a soil/vegetation mixture (c.f. snow for example where red reflectance is likely to be greater than near-infrared reflectance) [27]. Camera settings were as follows: 50 mm fixed focal length and high ISO and aperture priority mode to achieve relatively constant exposure with variable light conditions whilst minimizing any motion blurring. The LiDAR and digital camera were mounted 2.0 m above the ground while the GreenSeeker® was mounted 0.9 m above the ground, both being the respective heights that ensured sampling from the central rows of the plot, whilst avoiding the outermost rows. The data streams were geocoded with a wheel encoder and GPS/IMU system (0.2° and <1.0 m accuracy) fitted to the Phenomobile Lite. All data were captured on a tablet (Panasonic F7-G1 Toughpad 10.1-inch HD daylight readable display with powered docking station, Microsoft Windows) for later processing.

Data was captured on two separate occasions and referred to as “Event 1” and “Event 2.” For each event, the experiment was traversed with the Phenomobile Lite in serpentine fashion in the same direction of the sown rows, that is, in a north-south orientation (Figure 1). Plots were sampled in the same order at each time. For a given event, Phenomobile Lite measures occurred every hour commencing from 12:00 until 18:00 (local time); measurements then continued the following morning every hour from 07:00 to 12:00. In summary, Event 1 commenced 1-Aug-2017 (hourly measurements from 12:00 to 18:00) and concluded on 2-Aug-2017 (hourly measurements from 07:00 to 12:00). Event 2 commenced 17-Aug-2017 (hourly measurements from 12:00 to 18:00) and concluded on 18-Aug-2017 (hourly measurements from 07:00 to 12:00).

The phenological growth stage (GS) was recorded at each event using the scale of Zadoks et al. [28]. Specifically, Event 1 occurred at tillering GS and Event 2 at stem elongation GS.

#### 2.2.2. Data Processing

The LiDAR data was processed using a previously described custom-built processing pipeline (for details, refer to [14]), whereby the LiDAR data was first geocoded with the wheel encoder and GPS/IMU data. The LiDAR data was then manually segmented into experimental plots through a custom developed web interface. For a given plot, the outermost rows and a buffer at each end of about 0.5 m were excluded to avoid edge effects. Therefore, the dimensions of the sampling area for a given plot were approximately 1 m by 5 m. The wheel encoder and GPS/IMU data from the segmented LiDAR data were then used to assign the RGB images and GreenSeeker® NDVI data to each respective plot. In the case of RGB, an average of three images was assigned per plot; this ranged from one to five with a median value of three and 75th percentile value of four. For the GreenSeeker®, which sampled at 10 Hz, approximately 50 NDVI measures were assigned per plot (Phenomobile Lite operating speed was approximately 1 m per second).

**Figure 2:**
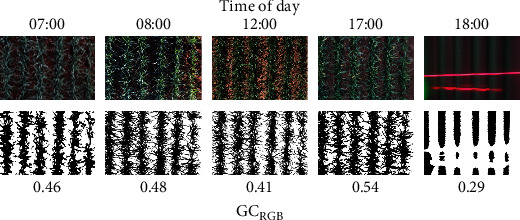
Example of RGB image processing, to derive GC_RGB_, under a range of light conditions with the time of day denoted at the top. For each time of day, the original RGB image is shown in the top row, and the resulting binary classification of the RGB image is shown in the lower row, together with the derived GC_RGB_. The images were extracted from the same experimental plot. The red lines in the 18:00 RGB image are from the LiDAR and GreenSeeker® sensors.

For the LiDAR, previously described algorithms (for details, refer to Figures 5, 6a,b in [14]) were then used for the classification of vegetation and soil to extract the following GC estimates from the LiDAR data: GC from red reflectance (GC_LiDAR_^RR^) whereby red reflectance less than five was classified as vegetation and GC from height (GC_LiDAR_^HT^) whereby height greater than 10 cm was classified as vegetation. The RGB images were analyzed using custom Python 2.7 code to determine the portion of green pixels for each image based on the previously reported vegetation index (VI) [11, 29, 30] (VI = (Green − Red)/(Green + Red)). A pixel was counted as green when the VI was greater than zero, and the proportion of green pixels in an image was reported as the GC (GC_RGB_). Examples of the RGB image processing for a range of light conditions are shown in Figure 2. The GC_RGB_ for each plot was determined as the mean GC_RGB_ of all the images belonging to the plot. Similarly, the NDVI for each plot was determined from the mean of the measurements belonging to the plot.

### 2.3. Statistical Analysis

Variance components were partitioned for each GC method, at each individual sampling time, using the SpATS package [31] (available from CRAN: https://cran.r-project.org/package=SpATS) in the R programming language (http://www.r-project.org). Spatial effects were modelled on a row and column basis by specifying the P-spline ANOVA (PSANOVA) algorithm, with the number of segments set to the respective number of rows and columns from the experimental design. The following factors were modelled as random effects: genotype, row, and column. The best linear unbiased predictors of genotype effects (BLUPs) were predicted from a fitted SpATS object. BLUPs are herein referred to as genotype means. Repeatability (*ρ*), sometimes called broad-sense heritability [32–34], was estimated: *ρ* = *σ*^2^_*g*_/(*σ*^2^_*g*_ + (*σ*^2^_*ε*_/*nrep*)), where *σ*^2^_*g*_ and *σ*^2^_*ε*_ are the genotypic and residual variances, respectively, and nrep is the number of genotype replicates in the experiment.

Correlations were estimated between variables using Pearson correlation analysis with the SciPy module [35] in Python 3.7 and statistically significant associations denoted: ^∗∗∗∗^*P* < 0.0001, ^∗∗∗^*P* < 0.001, ^∗∗^*P* < 0.01, and ^∗^*P* < 0.05. Figures were prepared using the matplotlib and seaborn Python modules [35].

The above analyses were completed to assess the reliability of the respective GC estimates. No attempt was made to determine or assess the absolute accuracy of a particular GC method, as the methods evaluated have been published previously (as cited in the introduction) and shown to be reasonably good indicators of GC. Moreover, when phenotyping large numbers of genotypes in breeding or prebreeding, the capacity to consistently discriminate (tested by repeatability analysis) and rank genotypes (tested by Pearson correlation analysis) is of arguably greater importance than the absolute accuracy of a particular method.

## 3. Results

### 3.1. Meteorological Conditions and Summary of Data

The hourly meteorological conditions for the two sampling events are shown in Table 1. On the afternoon of 1-Aug-2017, weather conditions were calm, with little to no wind, and clear with little cloud. The crop canopy was dry. Dew was evident on the leaves at 7:00 and 8:00, until about 9:00, on the morning of 2-Aug-2017; note that the dewpoint temperature (*T*_dew_) at these times was similar to the air temperature (*T*_air_) Table 1. No rain was recorded overnight. The sky was clear with little cloud, and light winds were present with gusts up to 13 kph.

For Event 2, which commenced at 12:00 on 17-Aug-2017, broken cloud was evident and conditions became slightly overcast in the afternoon. Wind gusts were up to 50 kph up to 17:00 and then down to 20 kph at 18:00. The crop canopy was dry from 12:00 until 18:00. The following morning on 18-Aug-2017, 0.6 mm of rain was recorded the previous night and the crop canopy was slightly damp until about 10:00. There was broken cloud and winds gusting up to 35 kph.

Possible discrepancies between solar radiation measured at Griffith NSW and the experimental site were investigated by comparing daily global solar exposure data (MJ · m^−2^), derived from satellite imagery (http://www.bom.gov.au), for the two sites. The solar exposure data is derived from satellite imagery processed by the Bureau of Meteorology from the Himawari series operated by the Japan Meteorological Agency and from GOES-9 operated by the National Oceanographic & Atmospheric Administration (NOAA) for the Japan Meteorological Agency. Differences were negligible (0.8%) for both days on Event 1 but greater for Event 2 (4.2% and 14.6% for 17 and 18-Aug, respectively) owing to the variable cloud cover.

Sunset occurred at 17:33 and 17:44 on 1-Aug-2017 (Event 1) and 17-Aug-2017 (Event 2), respectively. Sunrise occurred at 7:07 and 6:51 on 2-Aug-2017 (Event 1) and 18-Aug-2017 (Event 2), respectively.

**Figure 3:**
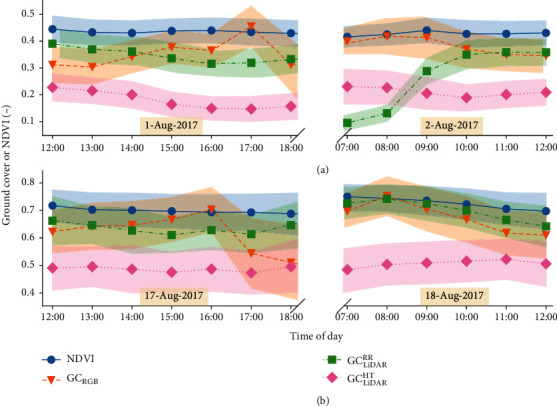
Summary of data from (a) Event 1 and (b) Event 2. Mean and standard deviation (shaded area) of normalized difference vegetation index (NDVI), ground cover from RGB camera (GC_RGB_), and LiDAR-derived ground cover from red reflectance (GC_LiDAR_^RR^) and height (GC_LiDAR_^HT^).

**Figure 4:**
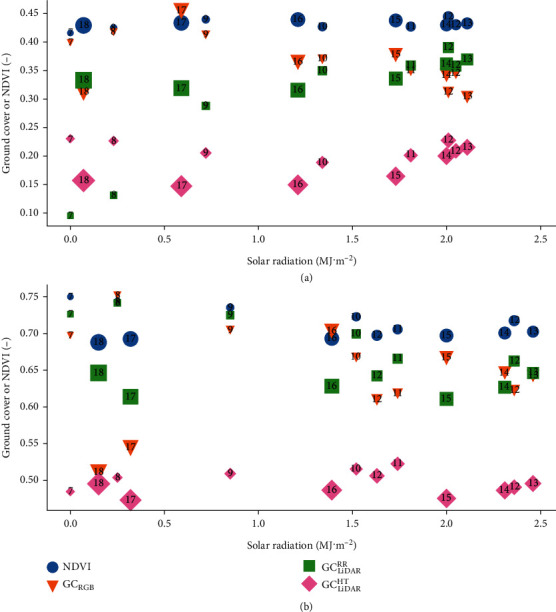
Mean ground cover or NDVI plotted against solar radiation, measured ca. 60 km northwest of the experiment site, as a relative indicator of the diurnal variation in daylight. (a) Event 1 and (b) Event 2. Each symbol corresponds to the mean value for a particular hourly measurement. The actual hourly measurement time is indicated by the number inside each symbol. The size of the symbol is scaled according to the hourly measurement time.

A summary of the GC data is presented in Figure 3 as the mean and standard deviation for each hourly sampling. To investigate the influence of solar radiation on the respective GC estimates, the mean GC for each particular hourly set of measurements was plotted against solar radiation (Figure 4).

The NDVI showed the least variation through time and across all hourly samplings, averaging 0.43 and 0.71, for Event 1 and Event 2, respectively. The possible exception being the contrast between the Event 2 hourly means at low solar radiation, whereby the readings at 07:00, 08:00, and 09:00 were notably greater than those at 18:00 and 17:00 (Figure 4).

In comparison to the NDVI, variation across the hourly samplings was greater for the RGB and LiDAR GC estimates (Figure 3). Specifically, GC_RGB_ increased from midday on 1-Aug-2017 and 17-Aug-2017 before decreasing at 17:00 and 16:00, respectively, presumably owing to the low light intensities in the evening. On the mornings of 2-Aug-2017 and 18-Aug-2017, the GC_RGB_ showed a general decreasing trend on both days. The influence of reduced daylight, as inferred from lower solar radiation, on GC_RGB_ was inconsistent (Figure 4). For example, for Event 1, there was a notable difference between GC_RGB_ at 17:00 and 18:00, while the GC_RGB_ at 07:00, 08:00, and 09:00 were consistent with each other. Additionally, for Event 2, the GC_RGB_ at 17:00 and 18:00 were similar but notably less than at 07:00 and 08:00. However, for both events, the GC_RGB_ showed a slight downward trend with increasing solar radiation from approximately 1.2 (MJ · m^−2^) onwards.

The LiDAR-derived GC estimate from red reflectance (GC_LiDAR_^RR^) was generally 0.15 greater than the estimate by the height method (GC_LiDAR_^HT^) from midday on both 1-Aug-2017 and 17-Aug-2017 until 18:00; both exhibited similar trends during these times (Figure 3). However, a different pattern was evident on the morning of 2-Aug-17, 07:00 to 09:00 in particular, whereby GC_LiDAR_^RR^ was greatly reduced from the previous day, possibly because of the dew evident at these times and high relative humidity (ca. 99% see Table 1). This pattern was not repeated for GC_LiDAR_^RR^ on the morning of 18-Aug-2017, where despite overnight rain of 0.6 mm, dew was not evident, and the relative humidity was comparatively less (ca. 80% see Table 1). The large discrepancy between GC_LiDAR_^RR^ estimates at low solar radiation for Event 1, where GC_LiDAR_^RR^ was significantly lower at 07:00 and 08:00 than at 17:00 and 18:00, was reversed for Event 2 (Figure 4), where GC_LiDAR_^RR^ at 17:00 and 18:00 was notably less than at 07:00 and 08:00.

In comparison to GC_LiDAR_^RR^, the trends for GC_LiDAR_^HT^ were generally stable and consistently lower than the other GC estimates, on the mornings of 2-Aug-2017 and 18-Aug-2017. Influence of the dew on GC_LiDAR_^HT^ on the morning of 2-Aug-17 (Event 1) was not as evident as that observed for GC_LiDAR_^RR^. However, under low solar radiation for Event 1, GC_LiDAR_^HT^ at 07:00 and 08:00 was notably greater than at 17:00 and 18:00 (Figure 4). This pattern was not repeated for Event 2.

The repeatability estimates (Table 2) were generally high across all GC measures, averaging 0.72, 0.7, 0.69, and 0.69 for NDVI, GC_RGB_, GC_LiDAR_^RR^, and GC_LiDAR_^HT^, respectively. The exceptions to this were GC_RGB_ at 18:00 on 1-Aug-17 and 17:00 on 17-Aug-17, where the repeatability estimates were 0.1 and 0.26, respectively.

### 3.2. Pearson Correlation Analysis between Measurements

**Figure 5:**
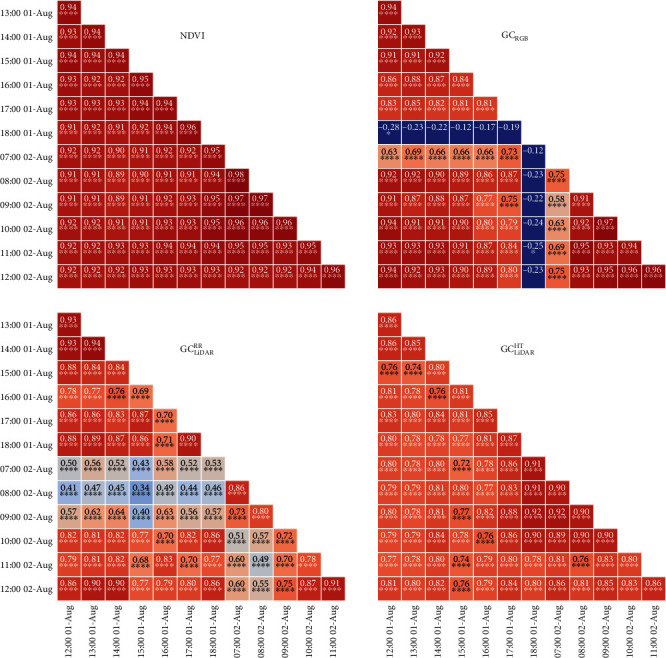
Event 1 intraclass correlations on individual plots (i.e., between individual samplings for a given ground cover estimate) for normalized difference vegetation index (NDVI), ground cover from RGB camera (GC_RGB_), and LiDAR-derived ground cover from red reflectance (GC_LiDAR_^RR^) and height (GC_LiDAR_^HT^). The time and date for each sampling is indicated.

**Figure 6:**
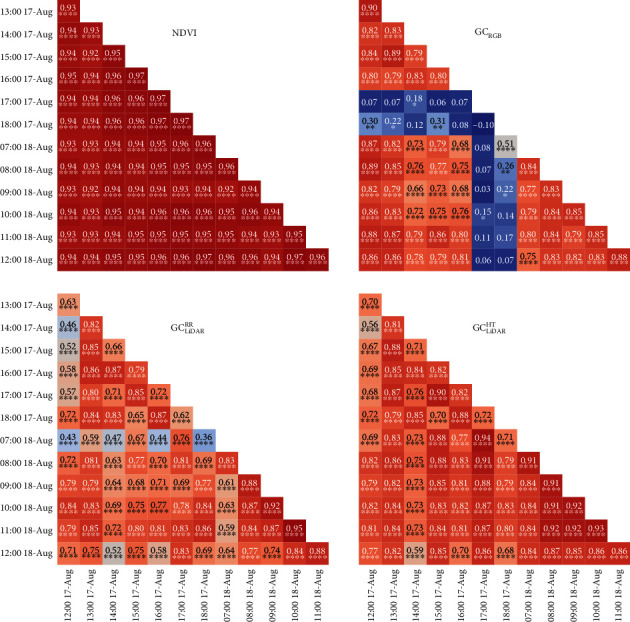
Event 2 intraclass correlations on individual plots (i.e., between individual samplings for a given ground cover estimate) for normalized difference vegetation index (NDVI), ground cover from RGB camera (GC_RGB_), and LiDAR-derived ground cover from red reflectance (GC_LiDAR_^RR^) and height (GC_LiDAR_^HT^). The time and date for each sampling is indicated.

**Figure 7:**
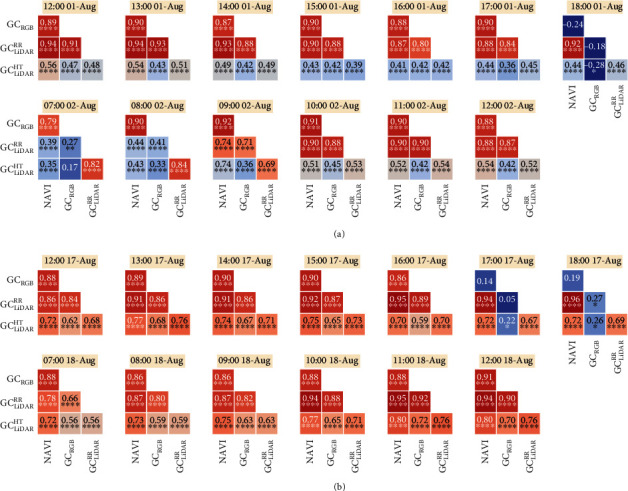
Phenotypic correlations on genotype means, for (a) Event 1 and (b) Event 2, between normalized difference vegetation index (NDVI), ground cover from RGB camera (GC_RGB_), and LiDAR-derived ground cover from red reflectance (GC_LiDAR_^RR^) and height (GC_LiDAR_^HT^). The time and date for each sampling is indicated.

Correlations on individual plots estimated across hourly measurements for a given GC estimate (e.g., NDVI) are referred to as intraclass correlations (ICCs) and are presented for Events 1 and 2 (Figures 5 and 6, respectively). In contrast, correlations on genotype means estimated between different GC estimates, for a given time, are referred to as phenotypic correlations and are shown for Events 1 and 2 in Figure 7.

The ICCs for NDVI were consistently high and significant (*P* < 0.0001) for both events, ranging from 0.89 to 0.98 (mean 0.93) for Event 1 (Figure 5) and from 0.92 to 0.97 (mean 0.95) for Event 2 (Figure 6). For GC_RGB_, ICCs with measurements on 18:00 on 1-Aug-2017 and 17:00 and 18:00 on 17-Aug-2017 were low and typically nonsignificant. Excluding these measurements, ICCs for GC_RGB_ were significant (*P* < 0.0001), ranging from 0.58 to 0.97 (mean 0.85) for Event 1 and from 0.66 to 0.9 (mean 0.81) for Event 2. The ICCs for GC_LiDAR_^RR^ were variable but significant (*P* < 0.0001) for all measurements on both events, ranging from 0.34 to 0.94 (mean 0.71) for Event 1 (Figure 5) and from 0.36 to 0.95 (mean 0.73) for Event 2 (Figure 6). In contrast, ICCs, for GC_LiDAR_^HT^ were high and significant (*P* < 0.0001) for both events, ranging from 0.72 to 0.92 (mean 0.82) for Event 1 and from 0.56 to 0.94 (mean 0.81) for Event 2.

Phenotypic correlations on genotype means were significant (*P* < 0.0001) for most of the hourly measurements, with the exception of GC_RGB_ measurements on 18:00 on 1-Aug-2017 and 17:00 and 18:00 on 17-Aug-2017 (Figure 7). Outside of these times, GC_RGB_ was significantly (*P* < 0.0001) correlated with NDVI, ranging from 0.79 to 0.92 across both events. GC_LiDAR_^RR^ was significantly (*P* < 0.0001) correlated with NDVI and GC_RGB_ for all samplings from 12:00 to 17:00 on 1-Aug-2017 (ranging from 0.80 to 0.94). However, correlations were lower from 07:00 to 09:00 on 2-Aug-2017 (ranging from 0.27 to 0.74) before increasing from 10:00 to 12:00 on 2-Aug-2017 (ranging from 0.87 to 0.90). Correlations between the same variables were generally high and significant (*P* < 0.0001) for Event 2, with the exception of the correlations with GC_RGB_ at 17:00 and 18:00 on 17-Aug-2017 and 07:00 on 18-Aug-2017. Phenotypic correlations involving GC_LiDAR_^HT^ were notably greater for Event 2 than Event 1. This improvement may have resulted from the increased mean crop height from 0.17, for Event 1, to 0.31 m, for Event 2.

## 4. Discussion

A diurnal time-course of GC measurements was derived from GreenSeeker® NDVI, RGB camera (GC_RGB_), LiDAR red reflectance (GC_LiDAR_^RR^), and LiDAR height (GC_LiDAR_^HT^). In contrast to GC_RGB_, GC_LiDAR_^RR^, and GC_LiDAR_^HT^, the NDVI values were highly stable through time (Figure 3) and highly correlated across repeated samplings (Figures 5 and 6). Moreover, the NDVI was not impacted by the varying light conditions or the presence of dew. Previous work has shown the GreenSeeker® NDVI particularly suited to discriminating crop canopies at early growth stages [18]. The presence of dew, on the morning of 2-Aug-2017, resulted in lower readings of GC_LiDAR_^RR^ (Figure 3). This is possibly due to the water, present on the leaves, interfering with the reflection of the LiDAR red-reflectance intensity signal and resulting in lower reflectance. However, there was no evidence to suggest that GC_LiDAR_^HT^ and GC_RGB_ were impacted by dew to the same extent. Nevertheless, that the presence of dew did not impact the NDVI was possibly because the dew formed predominantly on the leaves and not on the soil; the soil remained relatively dry. Previous work has shown that wet soils are generally less reflective in the red and near-infrared than dry soils (refer to Fig. 7.1 in [27]). Therefore, NDVI measured after heavy rainfall or irrigation may result in spurious readings.

The repeatability estimates (Table 2) were consistently high for all the GC measures, except for two GC_RGB_ sampling times (0.1 at 18:00 on 1-Aug-17 and 0.26 at 17:00 on 17-Aug-17) when the light was low. Additionally, with the exception of times when the light was exceedingly low, the phenotypic correlations between GC_RGB_ and NDVI were consistently high, ranging from 0.79 to 0.92. Notably, the GC_RGB_ repeatability estimate at 18:00 on 17-Aug-17 was high (0.82) when the correlations with NDVI (0.19), GC_LiDAR_^RR^ (0.27), and GC_LiDAR_^HT^ (0.26) were low (Figure 7). The inconsistent GC_RGB_ results, derived from the VI, at lower light conditions raise concerns for the application of this image analysis technique when light conditions are not ideal. That said, although GC_RGB_ was less stable across the different light conditions than the active NDVI, under adequate light conditions (midday and early afternoon) the GC_RGB_ often yielded higher repeatability estimates than the active NDVI. Thus, in adequate light conditions, the simple pixel-thresholding method used to derive GC_RGB_ was an effective method for quantifying the genetic variation in GC. This measurement mode contrasts with that used for the active NDVI, whereby the resulting NDVI is proportional to the difference between near-infrared and red reflectance from the area sampled. Despite these differences, under adequate light conditions, both methods were highly correlated.

The RGB camera is a passive light sensor, and as expected, there was evidence that the varying light conditions impacted on the consistency of the GC_RGB_ estimate from RGB (Figure 4). That is, the GC_RGB_ derived herein from the vegetation index was sensitive to the ambient light conditions. The GC_RGB_ appeared to decrease during the middle of the day, possibly because of over exposure from the sun, and decrease towards the end of the day because of the reduced light and under exposure. Despite previous studies reporting significant associations between RGB-derived GC and LAI [36], it is possible that more advanced image processing techniques may improve the stability of the GC estimates derived from the RGB images. One such technique is *K*-means clustering, where the pixels are clustered, for example, into soil, shadow, green, and brown leaves [37]. An extension of this concept has been developed to enable users to manually select the vegetation and background regions in a sample set of images to train a decision-tree-based pixel-segmentation model [15].

The LiDAR-derived GC estimates from height (GC_LiDAR_^HT^) were generally 0.15 to 0.2 less than the other GC estimates (Figure 3), indicating that the classification of ground from vegetation based on height may underestimate the proportion of vegetation. This may be due to the uneven ground resulting from cultivation and/or the presence of prostrate early leaves, which were often quite close (e.g., less than 10 cm above the ground). An alternative approach for classifying ground from the LiDAR point-cloud using machine learning was recently proposed [38] and may provide improved estimates of GC from LiDAR. Nevertheless, the phenotypic correlations between GC_LiDAR_^HT^ and the other GC estimates generally improved from Event 1 to Event 2 (Figure 7), as the average canopy height increased from 0.17 to 0.31 m, respectively. Previous work comparing GC estimates from NDVI and LiDAR, at nearly full canopy cover, indicated that the LiDAR (both GC_LiDAR_^RR^ and GC_LiDAR_^HT^) maintained sensitivity while the NDVI was saturated (refer to Figure 10 in [14]). Together, these provide support for further testing of GC_LiDAR_^HT^ at a high canopy cover, where the NDVI may saturate. Although the active NDVI was robust across sampling conditions of low light and dew in this study where GC was typically less than 0.75, GC_LiDAR_^HT^ may, subject to further testing, provide a potential alternative when GC is high (e.g., greater than 0.8). The latter is supported by evidence from previous studies showing that the GreenSeeker® NDVI can saturate at values greater than 0.8, often when the LAI is greater than 2.5 or 3 [18, 39].

The application of unmanned aerial vehicles (UAV) for phenotyping has greatly increased in recent years, aided by the availability of consumer-grade UAVs with suitable quality cameras. The weight limit of consumer-grade UAVs generally dictates the use of smaller cameras that rely on the ambient light conditions (passive sensors). They therefore require suitable light conditions and calm (low wind) weather for successful operation. That said, several studies have reported GC measures from UAV platforms using RGB [37, 40, 41] and multispectral [42, 43] cameras. The capacity to phenotype a large area in a short time is a key feature of UAVs; offering considerably greater throughput when compared to the Phenomobile Lite platform used herein. An alternative ground-based approach was recently presented whereby RGB cameras were mounted on a tractor-based system [44]. A defining feature of this application was that the plots were traversed at a right angle to the direction of seeding, thereby enabling simultaneous scanning of two plots. The latter reduced the travel distance by several times than when compared to traversing the plots in the direction of sowing and scanning each plot sequentially (as was the case for the Phenomobile Lite herein). Augmenting the tractor-based system presented by Walter et al. [44] with an active NDVI sensor would enable the assessment of GC early in the season across a range of light conditions and therefore times of day. This addition would increase the flexibility of the phenotyping platform and potentially improve the likelihood of adoption within plant breeding.

An additional consideration when using the active NDVI sensor is the need for close proximal sensing to ensure that the area sensed is constrained to the plot or plants of interest. In contrast to the RGB and LiDAR sensors, data from the active NDVI cannot be trimmed during post processing. Thus, the active NDVI is limited to ground-based platforms where the sensing height (and therefore the area sensed) can be readily controlled. With the capacity to trim RGB images, RGB cameras can be considered more flexible than the active NDVI sensor. Moreover, as discussed previously [45], RGB cameras are typically low-cost and afford the possibility of quantifying multiple traits from the same image (depending on the crop development stage).

The findings from this study have implications within the context of measuring GC for research and prebreeding studies and also for applications within a plant breeding program. In these applications, the capacity to reliably and repeatably phenotype GC, irrespective of weather conditions and time of day, is important. Moreover, particularly in the case of plant breeding, measurements may need to conform to a tight timeframe and can be opportunistic in nature. To this end, as highlighted in previous studies (e.g., [18]), the capacity to use an active sensor, such as the GreenSeeker® NDVI used herein, affords a high degree of flexibility for the user in terms of a broader range of acceptable light conditions and without being restricted to the middle of the day. Additionally, GC is a genetically complex phenotype of agronomic relevance with greater early GC likely to be of more benefit in wetter growing environments [6, 7]. Selection, therefore, either for higher or lower GC, is dependent on the capacity to consistently and precisely phenotype. With these applications in mind, the results in the present study support the use of active NDVI sensing from ground-based platforms, in preference to GC_RGB_ and the LiDAR methods (both GC_LiDAR_^RR^ and GC_LiDAR_^HT^), for estimating canopy GC when the LAI is below ca. 2.5 and the NDVI values are below ca. 0.8.

## Figures and Tables

**Table 1:** Meteorological conditions for the two sampling events, denoted Event 1 and Event 2. Hourly means of air (*T*_air_) and dewpoint temperature (*T*_dew_), relative humidity (RH), and solar radiation.

Event	Time and date	*T*_air_(°C)	*T*_dew_(°C)	RH (%)	Radiation (MJ · m^−2^)
Event 1	12:00 01-Aug-2017	15.1	5.1	51.4	2.0
	13:00 01-Aug-2017	16.2	4.5	46.2	2.1
	14:00 01-Aug-2017	16.5	2.7	39.7	2.0
	15:00 01-Aug-2017	16.5	1.8	37.3	1.7
	16:00 01-Aug-2017	16.2	2.8	40.6	1.2
	17:00 01-Aug-2017	13.4	3.0	49.7	0.6
	18:00 01-Aug-2017	11.7	2.9	55.2	0.1
	07:00 02-Aug-2017	3.6	3.4	99.0	0.0
	08:00 02-Aug-2017	5.7	5.2	96.7	0.2
	09:00 02-Aug-2017	8.8	4.8	76.5	0.7
	10:00 02-Aug-2017	11.0	3.8	61.3	1.3
	11:00 02-Aug-2017	13.1	4.1	54.7	1.8
	12:00 02-Aug-2017	14.9	4.0	48.2	2.0

Event 2	12:00 17-Aug-2017	16.3	4.3	45.1	2.4
	13:00 17-Aug-2017	16.7	4.6	44.9	2.5
	14:00 17-Aug-2017	16.9	4.3	43.3	2.3
	15:00 17-Aug-2017	16.8	4.1	43.0	2.0
	16:00 17-Aug-2017	16.4	3.9	43.4	1.4
	17:00 17-Aug-2017	15.1	4.2	48.4	0.3
	18:00 17-Aug-2017	14.0	4.1	51.1	0.1
	07:00 18-Aug-2017	7.2	4.3	82.3	0.0
	08:00 18-Aug-2017	8.2	4.5	77.5	0.2
	09:00 18-Aug-2017	9.4	5.0	74.1	0.8
	10:00 18-Aug-2017	10.6	5.1	68.9	1.5
	11:00 18-Aug-2017	11.3	4.4	63.0	1.7
	12:00 18-Aug-2017	12.1	3.7	56.7	1.6

**Table 2:** Repeatability estimates for the two sampling events, denoted Event 1 and Event 2, for canopy ground cover determined from normalized difference vegetation index (NDVI), RGB camera (GC_RGB_), and LiDAR-derived ground cover from red reactance (GC_LiDAR_^RR^) and height (GC_LiDAR_^HT^).

Event	Time and date	NDVI	GC_RGB_	GC_LiDAR_^RR^	GC_LiDAR_^HT^
Event 1	12:00 01-Aug-2017	0.68	0.70	0.69	0.70
	13:00 01-Aug-2017	0.60	0.68	0.59	0.52
	14:00 01-Aug-2017	0.58	0.69	0.65	0.56
	15:00 01-Aug-2017	0.64	0.65	0.58	0.58
	16:00 01-Aug-2017	0.66	0.63	0.48	0.65
	17:00 01-Aug-2017	0.65	0.72	0.62	0.74
	18:00 01-Aug-2017	0.71	0.10	0.71	0.77
	07:00 02-Aug-2017	0.76	0.73	0.61	0.77
	08:00 02-Aug-2017	0.75	0.82	0.66	0.79
	09:00 02-Aug-2017	0.72	0.79	0.65	0.79
	10:00 02-Aug-2017	0.71	0.74	0.70	0.73
	11:00 02-Aug-2017	0.68	0.77	0.62	0.63
	12:00 02-Aug-2017	0.63	0.71	0.69	0.62

Event 2	12:00 17-Aug-2017	0.79	0.81	0.66	0.59
	13:00 17-Aug-2017	0.70	0.78	0.75	0.70
	14:00 17-Aug-2017	0.71	0.80	0.72	0.67
	15:00 17-Aug-2017	0.73	0.76	0.74	0.75
	16:00 17-Aug-2017	0.77	0.69	0.75	0.63
	17:00 17-Aug-2017	0.76	0.26	0.77	0.77
	18:00 17-Aug-2017	0.76	0.82	0.81	0.75
	07:00 18-Aug-2017	0.76	0.85	0.63	0.80
	08:00 18-Aug-2017	0.78	0.77	0.75	0.77
	09:00 18-Aug-2017	0.78	0.67	0.71	0.70
	10:00 18-Aug-2017	0.79	0.74	0.75	0.68
	11:00 18-Aug-2017	0.80	0.77	0.80	0.69
	12:00 18-Aug-2017	0.74	0.73	0.77	0.73

## Data Availability

The processed data, for each individual plot, used to support the findings of this study are included within the supplementary information files. The primary data (RGB images and LiDAR data) used to support the findings of this study have been deposited in the CSIRO Data Access Portal (doi:10.25919/0xke-d287).

## Supplementary Materials

Supplementary 1 Data file S1. plot_level_data.csv.

Supplementary 2 Data file S2. about_plot_level_data.pdf.
