# Supplementary material for: Impact of Varying Light and Dew on Ground Cover Estimates from Active NDVI, RGB, and LiDAR
Source: Plant Phenomics. 2021 May 26;2021:9842178. doi: 10.34133/2021/9842178 (PMC8240513; doi:10.34133/2021/9842178)
Supplement: Supplementary 2 — Data file S2. about_plot_level_data.pdf. [file 9842178.f2.pdf]

About data

Table header in the data file	Description
PlotID	Unique identifier for experimental plot: PlotID = Column * 1000 + Row
Column, Row, Block	Experiment design
Entry	De-identified genotype
Date_Time	Date and time of sampling
HT	Crop height (meters), measured by LiDAR
GCRR	Fractional ground cover from LiDAR red reflectance (whereby red reflectance less than five was classified as vegetation)
GCHT	Fractional ground cover from LiDAR height (whereby height greater than 10 cm was classified as vegetation)
NDVI	Normalized Difference Vegetation Index (NDVI) measured by the commercially available GreenSeeker
GF	Fractional ground cover determined from RGB images as the portion of pixels from each image meeting a greenness criterion
